# Continued Dialogue about the Clinical Heterogeneity and Degenerative Pathology That Characterizes the Essential Tremors

**DOI:** 10.5334/tohm.1199

**Published:** 2026-03-30

**Authors:** Elan D. Louis

**Affiliations:** 1 Department of Neurology, University of Texas Southwestern Medical Center, Dallas, Texas, USA; 2 Peter O’Donnell Jr. Brain Institute, University of Texas Southwestern Medical Center, Dallas, TX, USA; 3 Department of Epidemiology, Peter O’Donnell Jr. School of Public Health, University of Texas Southwestern Medical Center, Dallas, TX, USA

**Keywords:** Essential tremors, Clinical, Intention tremor, Cerebellar, degeneration, heterogeneity

We would like to thank Dr. Khan [1] for commenting on our recent manuscript. We wish to make several responsive points. First, in the initial paragraph of our discussion, we had written as follows: “This observation is…also consistent with a cerebellar degenerative disease model.” We were careful not to use language such as “proves” or “supports”. Our intention was to point out that our observations *align with* the cerebellar degenerative model. This serves as a form of construct validity. Second, the authors incorrectly remark that “prior work has shown mixed and inconsistent evidence regarding structural cerebellar changes in essential tremor.” As pointed out previously [2,3,4,5,6], this is simply not the case. There are now more than 80 postmortem studies, spanning the period from 2005 to present, which demonstrate degenerative changes in the cerebellar cortex in ET [6,7]. Collectively, these studies have demonstrated in fine detail degenerative changes in the Purkinje cell as well as neighboring neuronal populations, with *more than thirty* such changes carefully documented [6,7]. Within this large contextual framework, there is a single study, from a decade ago, that (1) was not able to reproduce *one* of these pathological changes, and importantly, (2) did not assess any of the large number of other changes. That study as well as its null result have been critiqued repeatedly, and methodological features of the study make its validity questionable [2,3,4,5,6] (especially see extensive discussion in “PC death” section of Louis and Faust [2]). Third, they remark on the observed heterogeneity in longitudinal intention tremor trajectories. This is not surprising; in fact, lack of heterogeneity would have been surprising. Among other things, this heterogeneity is likely a function of the study’s modest time frame of 4.5 years (raising the likelihood of less stable and less consistent results), the use of an ordinal scale to measure intention tremor, ET’s known clinical heterogeneity [8], and the known heterogeneity of clinical expression of most neurological disorders. Indeed, there is a school of thought that ET should be renamed “the essential tremors” [9]. Fourth, the authors write “This variability suggests that progression of intention tremor is not uniform across individuals with essential tremor and may not represent an intrinsic or defining feature of the disorder in all cases.” Given the confusion in tremor nomenclature, further complicated by recent Consensus statements, it is probably best to stay clear of attempts to draw sharp boundaries with respect to what is or isn’t a “defining” or “intrinsic” feature, and best to focus on the careful collection of primary data. Fifth, we agree with Dr. Khan that future studies that incorporate clinical, biomarker and postmortem data are needed. That being said, these are difficult to achieve; the absence of an imaging biomarker for mild cerebellar degeneration is one limiting factor. The letter, however, is gratifying to read, as it focuses attention on the degenerative nature of ET, the complexity of its phenotype, and the likelihood that this is a family of diseases rather than a single etiologic-pathophysiologic-clinical entity.
